# Eosinophilic esophagitis in children and adolescents: a clinical practice guideline

**DOI:** 10.1186/s13052-025-02056-x

**Published:** 2025-07-23

**Authors:** Salvatore Oliva, Serena Arrigo, Matteo Bramuzzo, Fabio Cisarò, Emanuele Dabizzi, Giovanni Di Nardo, Paolo Gandullia, Massimo Martinelli, Maurizio Mennini, Fabio Monica, Lorenzo Norsa, Francesca Rea, Sara Renzo, Claudio Romano, Silvia Salvatore, Edoardo Vincenzo Savarino, Caterina Strisciuglio, Renato Tambucci, Carlo Calabrese, Paola De Angelis, On behalf of The Italian Society of Pediatric Gastroenterology Hepatology and Nutrition (SIGENP), The Italian Association of Hospital Gastroenterologists and Endoscopists (AIGO), The Italian Society of Gastroenterology (SIGE), and The Italian Society of Digestive Endoscopy (SIED), Marco Deganello Saccomani, Valeria Dipasquale, Enrico Felici, Cecilia Mantegazza, Paolo Orizio, Paolo Quitadamo, Alessandro Raffaele

**Affiliations:** 1 Pediatric Gastroenterology and Liver Unit, Maternal and Child Health Department, Sapienza University of Rome, Viale Regina Elena, 324 - 00161 Rome, Italy; 2 Pediatric Gastroenterology and Endoscopy Unit, IRCCS Istituto Giannina Gaslini, Genoa, Italy; 3 Institute for Maternal and Child Health - IRCCS “Burlo Garofolo”, Trieste, Italy; 4 Pediatric Gastroenterology Unit, Regina Margherita Children’s Hospital, Azienda Ospedaliera-Universitaria Città Della Salute E Della Scienza Di Torino, Turin, Italy; 5 Gastroenterology and Interventional Endoscopy Unit, Surgical Department, AUSL Bologna, Bologna, Italy; 6 Department of Neurosciences, Mental Health and Sensory Organs (NESMOS), Sapienza University of Rome, Pediatric Unit, Sant’Andrea University Hospital, Rome, Italy; 7 Department of Translational Medical Science, Section of Pediatrics, University of Naples Federico II, Naples, Italy; 8 Gastroenterology and Digestive Endoscopy Unit, Academic Hospital Cattinara, Trieste, Italy; 9 Deparment of Pediatrics, Vittore Buzzi Children’s Hospital, University of Milan, Milan, Italy; 10 Digestive Endoscopy and Surgery Unit, Bambino Gesù Children’s Hospital, Rome, Italy; 11 Gastroenterology and Nutrition Unit, Meyer Children’s Hospital, Florence, Italy; 12 Department of Human Pathology in Adulthood and Childhood “G. Barresi”Pediatric , Gastroenterology and Cystic Fibrosis Unit, University of Messina, Messina, Italy; 13 Department of Medicine and Technological Innovation, Pediatric Unit, Hospital “F. Del Ponte”, University of Insubria, Varese, Italy; 14 Department of Surgery, Oncology and Gastroenterology, University of Padua, Padua, Italy; 15 Department of Woman, Child, General and Specialistic Surgery, University of Campania “Luigi Vanvitelli,” Naples, Naples, Italy; 16 Department of Medical and Surgical Science, University of Bologna, Bologna, 40138 Italy

**Keywords:** Eosinophilic esophagitis, Food impaction, Children, Topical steroids, Pediatric endoscopy, PPI, Diet, Dysphagia, Guidelines, EoE

## Abstract

Eosinophilic esophagitis (EoE) is a chronic immune-mediated condition that affects the esophagus and is marked by the presence of eosinophils. This disease is becoming more common in children and adolescents and can result in symptoms like swallowing difficulties, food impaction and abdominal pain. Managing pediatric EoE requires a team effort including gastroenterologists, allergists and dietitians. Medical treatments may include topical corticosteroids, proton pump inhibitors, and elimination diets. Endoscopy plays a key role in the diagnosis, management and monitoring of the condition. The management of pediatric EoE is distinct from that of adult EoE, due to differences in anatomy, physiology and treatment options. Thus, it is recommended that children with EoE see a pediatric gastroenterologist when possible. However, adult gastroenterologists can also contribute to the management of pediatric EoE when a pediatric gastroenterologist is not accessible. A guideline for the management of pediatric EoE in Italy has been created by Italian Society of Gastroenterology, Hepatology and Nutrition (SIGENP) to encourage collaboration between adult gastroenterologists and pediatricians, and it has been endorsed by major adult gastroenterology Italian societies including AIGO, SIGE and SIED, highlighting the importance of collaboration in the diagnosis and management of pediatric EoE.

## Introduction

Eosinophilic esophagitis (EoE) is a chronic immune-mediated disease of the esophagus characterized by symptoms of esophageal dysfunction and eosinophil predominant inflammation in the esophagus [1]. This condition is increasingly recognized in children and adolescents, leading to symptoms like difficulty swallowing, food impaction, and abdominal pain [2].

EoE is primarily driven by Th-2 inflammatory mechanisms, often triggered by food antigens, and can cause variable symptoms, particularly in pediatric patients, such as growth failure, vomiting, regurgitation, dysphagia, and food bolus impaction [3]. Over the last decade, significant advances in the diagnosis and treatment of EoE have been reported [4].

Effective management of pediatric EoE requires a multi-disciplinary approach, involving gastroenterologists, allergists, and dietitians [5]. Medical treatment options may include topical corticosteroids (TCS), proton pump inhibitors (PPIs), and dietary interventions, including elimination diets. Endoscopy plays a crucial role in the diagnosis, management, and monitoring of disease activity and response to treatment.

While the first guideline for EoE was published in 2007 [6], several evidence-based updates and modifications have since been reported for both children and adults. The latest European pediatric guideline was updated in 2024 [7], and during the same period, an adult consensus provided additional information adapted to local feasibility, different drug regulations, and related costs [8, 9]. However, this document primarily focuses on the management of adult patients and does not specifically address the peculiarities and needs of the pediatric population.

Addressing this gap, the Italian Society of Pediatric Gastroenterology, Hepatology, and Nutrition (SIGENP) convened an expert panel of Italian pediatric gastroenterologists with expertise in various aspects of the disease to produce a comprehensive guideline for the management of pediatric EoE. The guideline outlines practical clinical approaches to treating pediatric patients with EoE.

Moreover, given that approximately one-third of endoscopic exams in Italy are performed by adult gastroenterologists [10], especially in emergency settings, SIGENP invited the three adult societies of gastroenterologists—the Italian Association of Hospital Gastroenterologists and Endoscopists (AIGO), the Italian Society of Gastroenterology (SIGE), and the Italian Society of Digestive Endoscopy (SIED)—to participate and endorse this document.

It is important to note that the management of pediatric EoE can differ significantly from that of adult EoE [11]. Pediatric gastroenterologists are specifically trained to deal with the unique challenges posed by this condition in children and adolescents, including differences in anatomy, physiology, and treatment options. As such, it is recommended that children and adolescents with EoE be seen by a pediatric gastroenterologist whenever possible. However, adult gastroenterologists can also play a vital role in managing this condition, especially in cases where a pediatric gastroenterologist is not available. This guideline was established to encourage collaboration between adult gastroenterologists and pediatricians in Italy.

Unlike other published guidelines, the proposed guideline is intended to assist Italian healthcare providers in all aspects of the diagnostic process and the relative management of pediatric patients with eosinophilic esophagitis. It aims to provide a comprehensive resource for healthcare professionals to effectively diagnose and manage pediatric EoE cases in Italy.

## Methods

The SIGENP Endoscopy Working Group (WG), in collaboration with the three Italian societies of adult Gastroenterologists (AIGO, SIGE, and SIED), commissioned a panel of experts to develop a comprehensive clinical practice guideline. The objective was to support general pediatricians, pediatric gastroenterologists, and adult endoscopists in effectively managing children and adolescents with EoE.

The guideline coordinators (SO and PDA) identified ten main tasks for the panel: 1) definition; 2) epidemiology; 3) diagnosis; 4) comorbidities; 5) endoscopy; 6) histology; 7) motility assessment; 8) therapy; 9) diet; and 10) transition and quality of life.

To formulate key questions, the panel followed the PICO format and voted (13). A comprehensive literature search on English-written articles was conducted using PubMed/EMBASE, with no time limits and relevant MeSH terms. The panel engaged in regular conference calls, web-based exchanges, and two intermediate meetings.

Using the GRADE system as general guidance, the levels of evidence and recommendations were established for each part of the guideline. Remote meetings were conducted twice for the working parties to review and reach consensus on the statements. Each statement was revised until agreement was reached. The panel then voted on all recommendations and practice points using a web-based voting platform. A 5-point scale (1: strongly disagree; 2: disagree; 3: neutral; 4: agree; 5: strongly agree) was employed for rating statements, and a consensus was reached if over 80% of participants agreed (grade 4–5). Statements without consensus were revised and voted on again. In cases of discrepancies or conflicts in the available evidence, consensus was reached through a structured iterative process. The document was revised based on received comments, followed by a second round of electronic voting and further revisions. Whenever possible, statements and recommendations were evidence-based. Pediatric data were prioritized whenever available; in the absence of pediatric studies, adult data were considered, and when no direct evidence was available, consensus among panelists was sought based on clinical experience and expert opinion.

Evidence was downgraded following standard GRADE criteria, considering risk of bias, inconsistency, indirectness, imprecision, or publication bias, and could be upgraded in the presence of large effects, dose–response relationships, or plausible confounding reducing observed effects. The threshold for consensus was set at ≥ 80% agreement (Likert scale scores 4–5), a level chosen to balance methodological rigor with practical feasibility across a large, multidisciplinary panel. Alternative thresholds (70% and 90%) were discussed but dismissed to avoid either excessively loose or unattainably strict consensus requirements.

The guideline incorporates both recommendations and “practice points” that reflect common practices in situations where evidence is lacking. Weaker recommendations are indicated by phrases such as “we suggest,” while stronger recommendations are stated as “we recommend.” It is important to read recommendations in the context of qualifying comments in the accompanying text.

To draft the initial manuscript, each working group provided a summary of background evidence for the statements, which was compiled by SO. The manuscript was circulated among the consensus group for revisions before submission for publication.

The clinical practice guideline serves as a comprehensive resource for healthcare professionals in Italy, guiding them in the management of pediatric patients with eosinophilic esophagitis effectively.

### Executive summary

This executive summary highlights the key clinical recommendations from these guidelines on pediatric EoE. It is intended to support general pediatricians and primary care providers in the prompt diagnosis and management of EoE, with a focus on practical, evidence-based strategies.

• Definition:
  EoE is a chronic, immune-mediated esophageal disease characterized by esophageal dysfunction symptoms and eosinophilic inflammation (≥ 15 eosinophils/high-power field). PPI trials are no longer required for diagnosis.
• Epidemiology:
  The incidence and prevalence of EoE are increasing, estimated at up to 1 in 1,000 in Western countries. Males and children with atopic diseases are at higher risk.
• Clinical Presentation:
  - ◦ Infants: Vomiting, reflux-like symptoms, feeding refusal, failure to thrive.
  - ◦ Older children/adolescents: Dysphagia, food impaction, chest pain.
• Diagnosis:
  - ◦ Upper GI endoscopy with ≥ 6 esophageal biopsies (from different sites) is mandatory.
  - ◦ Allergy tests should assess for comorbid atopic diseases but are not reliable to guide food elimination.
  - ◦ PPIs should be stopped 3–4 weeks before biopsies.
• Therapeutic Goals:
  Improvement across all disease domains: symptoms, endoscopy, and histology.
• First-Line Therapies:
  Selection should be guided by clinical presentation, endoscopic phenotype, histology, and patient/family preference:
  - ◦ PPI therapy (2 mg/kg/day) is the preferred first-line treatment in mild cases.
  - ◦ Topical corticosteroids (swallowed budesonide or fluticasone) are effective, particularly for inflammatory forms.
  - ◦ Dietary therapy (starting with cow’s milk elimination) can be considered, especially in atopic children.
  - ◦ Biologic therapy (dupilumab) is an option for severe, fibrotic disease or when other treatments fail.
• Follow-Up:
  - ◦ Endoscopy with biopsies is recommended 8–12 weeks after therapy initiation or adjustment.
  - ◦ Long-term monitoring (every 1–2 years) is necessary, even if symptoms resolve, to prevent fibrosis.
• Special Considerations:
  - ◦ Esophageal dilation may be needed for strictures.
  - ◦ Psychological support is important for children with EoE and psychiatric comorbidities.
• Referral:
  Pediatric gastroenterologist involvement is strongly recommended. Collaboration with allergists and dietitians is advised.

### Definition

Q1: What is the definition of Eosinophilic Esophagitis?

Statement 1

EoE is an immune-antigen-mediated chronic condition characterized by symptoms of esophageal dysfunction, a predominant eosinophilic infiltration in the esophageal mucosa, and the absence of alternative causes of eosinophilic inflammation.

[high quality of evidence]

[Vote result: Strongly agree: 83%; agree: 17%, neutral: 0%, disagree: 0%, strongly disagree: 0%]

Practice point

PPI trials are no longer required to diagnose EoE

Summary of evidence:

Historically, distinguishing between EoE and gastroesophageal reflux disease (GERD) has posed challenges, as both conditions can lead to esophageal eosinophilia [12]. Initially, the diagnostic approach for EoE required either a 24-h pH-monitoring or a 2-month therapeutic trial with high-dose PPIs to rule out GERD as the underlying cause of symptoms and eosinophilic infiltrates before confirming EoE [13].

Previous pediatric and adult guidelines mandated an 8-week trial of high-dose PPIs and a follow-up endoscopy to exclude PPI-responsive eosinophilia (PPI-REE) before confirming the diagnosis of EoE [13, 14]. However, a significant change occurred in 2017 when patients with esophageal dysfunction and eosinophil-predominant esophageal inflammation without any alternative diagnosis were considered to have EoE [15, 16]. According to these new definitions, patients with esophageal dysfunction and eosinophilic infiltrates can be diagnosed with EoE even if they achieve clinical and histologic remission on PPI therapy, removing the need for additional diagnostic trials with PPIs. As a result, PPIs are now considered a treatment option rather than a diagnostic criterion.

This shift in approach has simplified the diagnostic process for EoE, allowing healthcare professionals to promptly identify and manage the condition.

### Epidemiology

Q 2.1: What are the current incidence and prevalence of EoE in children?

Statement 2.1.1

A continuous increase in EoE incidence and prevalence rates has been reported both in children and adults over the last 2 decades*.*

[moderate quality of evidence]

[Vote result: Strongly agree: 91%; agree: 9%, neutral: 0%, disagree: 0%, strongly disagree: 0%]

Statement 2.1.2

Currently, there is not enough evidence to determine the true incidence and prevalence in the pediatric Italian population.

[low quality of evidence]

[Vote result: Strongly agree: 48%; agree: 52%, neutral: 0%, disagree: 0%, strongly disagree: 0%]

Practice point

The current estimated incidence and prevalence in Western countries are up to 20 per 100,000 people per year and 1 per 1,000 people, respectively.

Summary of evidence:

In the last two decades, there has been a significant and continuous increase in the incidence and prevalence of EoE reported in various studies [17, 18]. This increase in prevalence and incidence of EoE seems to be outpacing the improvement in disease recognition, and a plateau has not yet been reached [19, 20]. In 2016, Arias and colleagues conducted the first systematic review of EoE and found an overall incidence of 3.7 per 100,000 person-years and a prevalence range of 22.7 per 100,000 person-years, with a trend of increasing incidence over successive years [21]. The prevalence of EoE appeared to be higher in adults than in children (43.4; 95% CI: 22.5–71.2 vs. 29.5; 95% CI: 17.5–44.7, respectively), and in American studies compared to European studies [21]. Although not statistically significant (*p* = 0.46), there was an observed increase in EoE prevalence rates, rising from 17.9 cases per 100,000 inhabitants/year (95% CI 7.4–32.9; I2 = 98.3%) in studies carried out before 2008 to 26.3 cases per 100,000 (95% CI 12.3–45.5; I2 = 99.9%) in research carried out in 2008 or later.

More recently, following the inclusion of PPI-responsive patients in the EoE spectrum, Navarro and colleagues updated the meta-analysis, including 27 population-based studies, with 14 studies reporting EoE prevalence in children [22]. The overall prevalence of EoE in children was 34.4 cases per 100,000 inhabitants (95% CI, 22.3‐49.2; I2 = 99.7%), with no significant differences between the United States and Europe (38.3; 95% CI 23.7‐56.4 and 41; 95% CI, 3.2‐121.1, respectively) [22]. Ten pediatric studies evaluating the incidence of EoE were included in the analysis, and the overall incidence rate was 6.6 per 100,000 person-years (95% CI 3–11.7; I2 = 99.8%). The pooled prevalence and incidence of EoE in adults were 42.2 (95% CI, 31.1‐55) and 7.7 per 100,000 (95% CI, 1.8‐17.8), respectively, which were higher than in children [22].

Although clear epidemiological data are not available for Italy, the current estimated incidence and prevalence may be similar to those in other Western countries, with rates of up to 20 per 100,000 people per year and 1 per 1,000 people, respectively. [23].

Based on the available evidence, clinicians should be aware of the increasing incidence and prevalence of EoE and consider these factors when evaluating and managing patients with suspected or diagnosed EoE.

Q 2.2: Are there environmental factors associated to the development of EoE in children?

Statement 2.2.1

The risk of developing EoE is reported to be 2–3 times higher in males than in females.

[high quality of evidence]

[Vote result: Strongly agree: 88%; agree: 12%, neutral: 0%, disagree: 0%, strongly disagree: 0%]

Statement 2.2.2

Children with EoE are at a higher risk of having concomitant atopic manifestations such as asthma, allergic rhinitis, and eczema compared to children without EoE.

[high quality of evidence]

[Vote result: Strongly agree: 96%; agree: 4%, neutral: 0%, disagree: 0%, strongly disagree: 0%] Statement 2.2.3

Current evidence suggests that early life exposures, such as formula feeding and early prescription of PPIs and antibiotics, are associated with the subsequent development of pediatric EoE.

[low quality of evidence]

[Vote result: Strongly agree: 48%; agree: 42%, neutral: 10%, disagree: 0%, strongly disagree: 0%]

Summary of evidence

Several studies in Europe and the US have consistently reported a higher prevalence of EoE in males than in females [24, 25]. In a systematic review with a meta-analysis conducted by Arias and colleagues in 2016 [21] and updated in 2019 [24], male patients with EoE had a pooled prevalence of 72.1 (95% CI, 41.3–111.5; I2 = 99.9%) per 100,000 inhabitants, while the prevalence in females was 29.4 (14.8–48.8; I2 = 99.9%) per 100,000 inhabitants. The odds ratio for EoE in males compared to females was 2.22 (95% CI: 2–2.46). Recent data from the PEER registry, which is the pediatric European Registry of the European Society of Gastroenterology, Hepatology, and Nutrition (ESPGHAN), also confirmed a male predominance, with 76% of pediatric EoE cases occurring in males [2, 26].

Children with EoE often have concomitant atopic manifestations, such as asthma, allergic rhinitis, and eczema [27–30]. A meta-analysis found that EoE patients had a significantly higher risk of these conditions compared to the control population, with odds ratios ranging from 2.8 to 5.1 [31]. Recent data from the PEER registry also confirmed that two-thirds of children with EoE had a history of atopy [2, 26], indicating that EoE children are at a higher risk of concomitant atopic manifestations. However, the lack of studies properly comparing EoE children with healthy controls and the lack of standardization of atopy definition have limited our understanding of the role of atopy in EoE pathogenesis.

Several retrospective studies have investigated whether early life exposures, such as cesarean section, preterm birth, NICU admission, formula feeding, and early prescription of PPIs and antibiotics, are associated with the subsequent development of pediatric EoE [27, 30, 32, 33]. These studies suggest that antibiotic use in infancy may be associated with a higher risk of EoE, and that cesarean delivery, preterm birth, and formula-only or mixed feeding also have trends toward increased odds of developing EoE. The use of PPIs, histamine-2 receptor antagonists, and antibiotics was also associated with EoE. Prematurity and early manifestations of atopic disease, such as milk protein allergy and eczema, were related to an increased risk of EoE. A recent study by Jensen and colleagues found a positive association between EoE and prenatal, intrapartum, and infancy factors [33]. While possible early microbiome perturbations have been hypothesized as a causative agent [34, 35], the lack of well-defined prospective studies precludes definitive conclusions.

### Diagnosis

Q 3.1 How do the symptoms of EoE typically manifest in affected individuals?

Statement 3.1

In infants and younger children, EoE commonly presents with reflux-like symptoms, vomiting, abdominal pain, food refusal, and failure to thrive. Dysphagia, food impaction, and non-swallowing associated chest pain are the most reported symptoms in older children and adults with EoE. *[high quality of evidence].*

[Vote result: Strongly agree: 98%; agree: 2%, neutral: 0%, disagree: 0%, strongly disagree: 0%]

Summary of evidence

EoE can manifest at any age, although it usually begins in childhood. The clinical manifestations vary depending on the age at presentation. Infants and young children typically present with nonspecific symptoms such as reflux-like symptoms, vomiting, nausea, abdominal pain, food refusal, or failure to thrive [2, 12, 36–38]. Dysphagia and food impaction episodes become more common in school-aged children and adolescents, similar to adult patients. Heartburn, regurgitation, and chest pain are also associated symptoms in these age groups.

Q 3.2 What are accepted diagnostic criteria for diagnosis of EoE in pediatric patients?

Statement 3.2

The diagnosis of eosinophilic esophagitis (EoE) is established by the presence of eosinophilic infiltration of the esophageal mucosa, typically with more than 15 eosinophils per high-power field (using a standard size of 0.3 mm^2^), based on biopsy specimens obtained from at least two different locations in the esophagus, usually the distal and proximal parts. The diagnosis requires the presence of clinical symptoms of esophageal dysfunction, such as dysphagia or food impaction, and the exclusion of other causes of esophageal eosinophilia.

[high quality of evidence]

[Vote result: Strongly agree: 90%; agree: 10%, neutral: 0%, disagree: 0%, strongly disagree: 0%]

Recommendation 3.2

We recommend that upper gastrointestinal (GI) endoscopy with multiple biopsies of the esophagus should be performed to diagnose children with suspected EoE. At least six biopsies should be obtained, with special attention to visible lesions and samples taken from the proximal, mid, and distal segments of the esophagus.

[high quality of evidence, grade of recommendation: strong]

[Vote result: Strongly agree: 88%; agree: 12%, neutral: 0%, disagree: 0%, strongly disagree: 0%]

Practice point.

PPIs should be discontinued at least 3–4 weeks before biopsy collection.

Summary of evidence

The main diagnostic criterion for EoE involves biopsies that reveal a peak value of ≥ 15 eosinophils per high power field (hpf). Originally, this cut-off was determined using analogue optical microscopes that had low and high-power magnification. Since digital optical microscopy is now used in many laboratories, the peak eosinophil value should be reported as ≥ 15 per 0.3 mm2. According to the International Consensus guidelines, 15 eosinophils/hpf should be equivalent to 60 eosinophils per square mm [15, 16]. However, the historical threshold of 15 eosinophils per 0.3 mm2 is still widely used, especially given the patchy distribution of eosinophilic infiltration in the esophageal mucosa. It is important to note that multiple biopsies are associated with higher diagnostic accuracy [12].

**Fig. 1:**
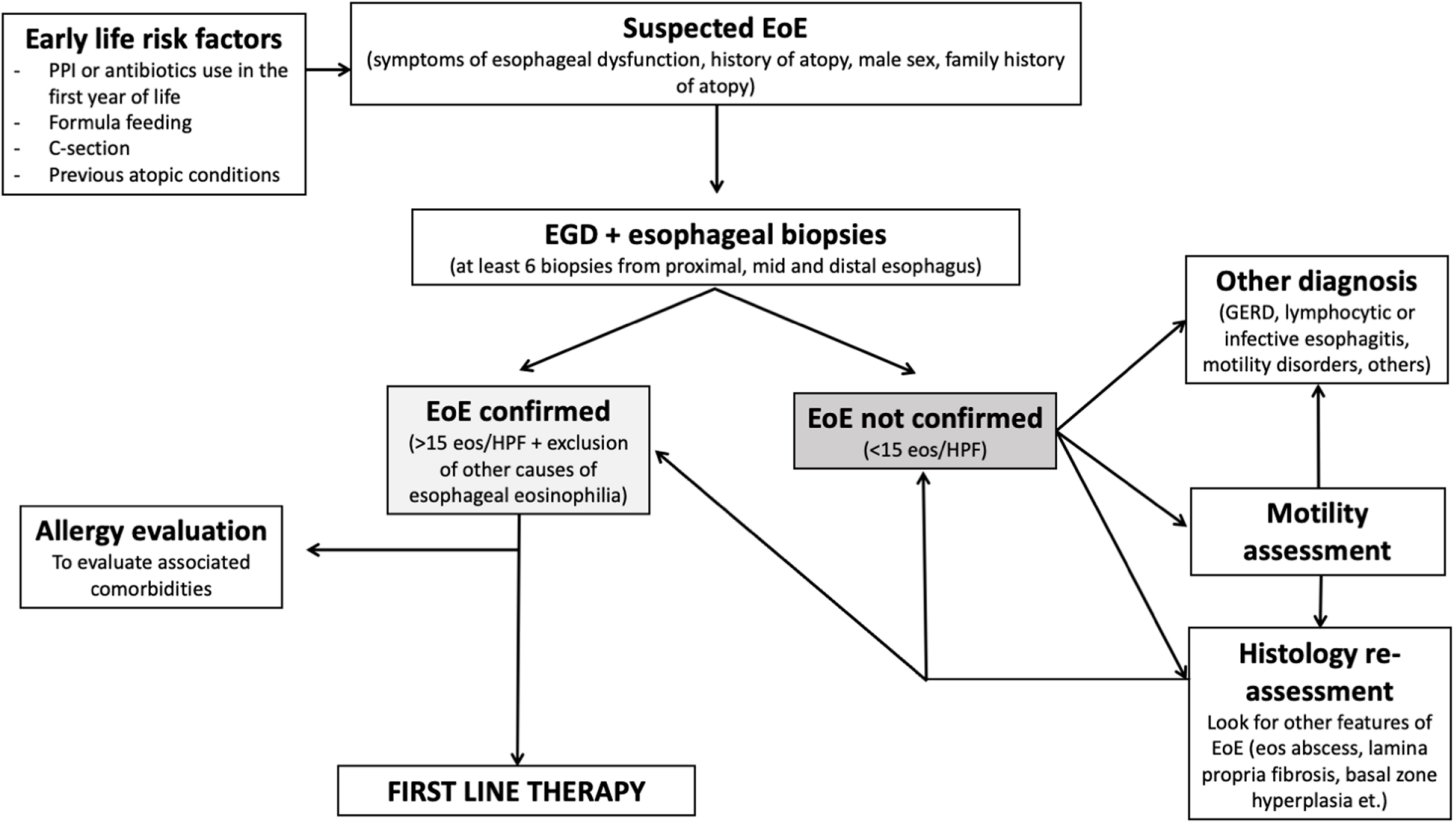
Diagnostic Algorithm for Pediatric EoE. Stepwise approach to diagnosing EoE, including symptom assessment, endoscopy with biopsies, and exclusion of alternative causes

Other conditions, such as eosinophilic gastrointestinal disease, lymphocytic esophagitis, infectious esophagitis, connective tissue disorders, vasculitis, hypereosinophilic syndrome, Crohn’s Disease, and celiac disease, should be excluded to ensure an accurate diagnosis [39, 40]. In the presence of non-esophageal gastrointestinal symptoms, EGIDs should always be ruled out by collecting biopsies also from the stomach and/or duodenum [15, 40]. The diagnostic algorithm is presented in Fig. 1.

Q 3.3 What is the value of allergy testing in EoE?

Recommendation 3.3.1

We suggest that allergy tests should be performed at the time of diagnosis to evaluate potential concomitant atopic comorbidities that may need to be addressed in the management of the patient.

[moderate quality of evidence, grade of recommendation: weak]

[Vote result: Strongly agree: 58%; agree: 42%, neutral: 0%, disagree: 0%, strongly disagree: 0%]

Recommendation 3.3.2

We recommend against the use of allergy tests to identify trigger foods for EoE, as there is no reliable correlation between the presence of specific IgE antibodies and EoE.

[high quality of evidence, grade of recommendation: strong]

[Vote result: Strongly agree: 84%; agree: 16%, neutral: 0%, disagree: 0%, strongly disagree: 0%]

Summary of evidence:

Current data do not support the use of IgE testing (via Skin Prick Test or serum) and Atopy Patch Test to identify triggering food allergens in EoE [41–46] (40–46). The largest meta-analysis to date, which included data from 1317 patients with EoE (1128 children and 189 adults) who received different dietary treatments, found that the combined effectiveness (histology and symptoms) of targeted diet did not reach 50%, and there was a wide range of variation in remission rates reported in individual studies [47].

Novel strategies based on IgE tests, such as component analysis [48, 49], have also not been able to predict food triggers for EoE. These findings support the notion that EoE is characterized by a non-IgE-mediated food hypersensitivity, and as such, the use of IgE-based tests should be limited to the management of IgE-mediated allergy [50–53].

Recent studies [54, 55] have suggested a possible association between EoE and elevated levels of total IgG4 and food-specific IgG4 in serum or the esophagus. These findings suggest that IgG4 may be involved in EoE pathogenesis, potentially via the blockage of IgE-allergen binding by food-immunoreactive IgG4. However, there are few evidence on the efficacy of an IgG4-tailored diet. Therefore, allergy tests are not recommended to identify trigger foods in EoE, but they should be performed at diagnosis only to evaluate potential concomitant atopic comorbidities.

Q 3.4 Are there any non- or minimally invasive biomarkers that are useful for diagnosing or managing treatment in children with EoE?

Statement 3.4

Currently, there are no minimally invasive or non-invasive tests with sufficient accuracy for routine use in diagnosing or managing childhood EoE. However, several biomarkers are being investigated for their potential to identify disease activity and response to treatment, which may be promising avenues for future research.

[moderate quality of evidence]

[Vote result: Strongly agree: 68%; agree: 22%, neutral: 10%, disagree: 0%, strongly disagree: 0%]

Practice point:

Less invasive techniques, such as trans-nasal endoscopy, esophageal string test, and esophageal sponge test, may be an option in specialized referral centers with expertise in performing these procedures.

Summary of evidence:

Since 2013, multiple studies have assessed both non- and minimally invasive biomarkers, as well as non-endoscopic techniques to access esophageal tissue [56–58]. Studies have demonstrated statistically significant differences in the mean or median values of these biomarkers between patients and controls, or untreated vs. treated patients, however, most showed a significant overlap between the groups precluding their use as accurate biomarkers. Nevertheless, a systematic review highlighted the biggest weakness of most of these studies, which is the lack of atopic controls since the majority of EoE patients have additional allergic conditions which must be considered when using biomarkers, which are mainly associated with the immune system [59].

While biomarkers are still lacking, non-endoscopic techniques seem promising. The esophageal string test has shown good accuracy in prospective pediatric [57, 60] or combined pediatric/adult studies [61], and the Cytosponge [62, 63] has been successfully implemented in adult studies. These techniques need to be further validated in general, and more specifically in children, not only for their efficacy but also for tolerability.

Unsedated transnasal endoscopy (TNE) has been proposed as a minimally invasive alternative to conventional sedated endoscopy [63, 64]. This technique is safe, less expensive than sedated EGD, and can be performed with topical anesthesia, using audio or visual distractions to reduce patient discomfort. TNE has been studied in the treatment of pediatric EoE and was found to be safe, provided adequate biopsy samples, was cost-effective, and required less office time than standard endoscopy. While 85% of parents preferred it over sedated endoscopy, only 52% of pediatric patients did [63]. This technique requires further validation in larger cohorts. However, several referral centers in Italy have already begun using TNE due to its safety and tolerability.

### Comorbidities

Q 4.1: Are motility disorders associated to EoE in children?

Statement 4.1

While it is known that EoE can be associated with impaired esophageal motility, there is currently no evidence to suggest that primary motility disorders can cause EoE in children.

[low quality of evidence]

[Vote result: Strongly agree: 48%; agree: 52%, neutral: 0%, disagree: 0%, strongly disagree: 0%]

Recommendation 4.1.1

We suggest performing barium esophagogram and esophageal manometry to identify coexisting esophageal motility disorders in patients with persistent EoE symptoms.

[low quality of evidence, grade of recommendation: weak]

[Vote result: Strongly agree: 84%; agree: 16%, neutral: 0%, disagree: 0%, strongly disagree: 0%]

Recommendation 4.1.2

We suggest considering endoscopy with esophageal biopsies to rule out EoE in patients with motility disorders and persistent symptoms.

[moderate quality of evidence, grade of recommendation: weak]

[Vote result: Strongly agree: 88%; agree: 12%, neutral: 0%, disagree: 0%, strongly disagree: 0%]

Summary of Evidence.

Motility disorders, such as achalasia, hypercontractile or hypocontractile esophagus, and altered lower esophageal sphincter pressure, have been identified in adult patients with EoE. Limited data is currently available on the prevalence of these disorders in children [65–67]. The pathogenic mechanisms of motility impairment in EoE involve eosinophilic infiltration, mast cell increase, histamine release, activation of acetylcholine pro-inflammatory mediators, and neuroactive eosinophil products that induce impaired esophageal peristalsis. Eosinophilic cationic protein may destroy myenteric neurons, inhibiting the relaxation of the lower esophageal sphincter. Conversely, esophageal stasis can promote inflammation and eosinophil recruitment. Whether the motility disorder is primary or secondary to EoE needs further clarification [68].

EoE and eosinophilic esophageal infiltration have been reported in a variable proportion of patients with achalasia, suggesting a possible cause-effect relationship [66, 67]. Improvement and normalization of esophageal motor function have been reported after EoE treatment. However, the relationship between EoE and motility disorders remains complex, as the reduction of eosinophilic infiltration documented after achalasia treatment in selected subjects suggests a bidirectional interaction [69]. In a recent study on 109 EoE adult patients, 38% of them showed esophageal abnormalities on high resolution manometry, and achalasia was significantly associated with female gender, longer diagnostic delay, and abnormal esophagogram. Clinical features and endoscopic findings were not significantly different between patients with EoE with or without achalasia and obstructive motor disorders [70].

Q 4.2: How does allergy relate to the development of EoE in children?

Statement 4.2

The risk of developing EoE is higher in atopic individuals, especially those with a history of food allergy.

[moderate quality of evidence]

[Vote result: Strongly agree: 74%; agree: 26%, neutral: 0%, disagree: 0%, strongly disagree: 0%]

Practice Point

Consider the potential triggering effect of aeroallergens and oral immunotherapy in the management of EoE in atopic patients.

Summary of Evidence

Allergy has long been considered a crucial factor in EoE due to the overlap in clinical manifestations, family history of allergy, and positive allergy tests in about 50% of EoE cases. Healing of esophagitis after a food elimination diet in 70–90% of pediatric patients also supports the role of allergy in EoE [71]. Genetic, environmental, and immunologic factors are shared between allergy and EoE [72]. An increasing number of studies show that genetic predisposition is associated with different EoE phenotypes and the risk of sensitization to aeroallergens or food allergens. Genetic loci are mainly involved in T-helper 2 type inflammation and epithelial barrier function and integrity [73]. For instance, individuals with a specific genetic predisposition (TSLP risk allele) showed increased food allergen triggers, regardless of prior atopic disease, and a low response to a single-food elimination diet if homozygous for the risk allele [73].

EoE patients present a striking prevalence of atopic conditions, such as atopic dermatitis, IgE-mediated food allergy, allergic rhinitis, and asthma, with rates varying from 10–25% for atopic dermatitis and IgE-mediated food allergy to 30–90% for allergic rhinitis and 25–50% for asthma [72, 74]. In children and adolescents, the prevalence of EoE in subjects with food allergy was found to be 100 times higher than in the control population (4.7% vs. 0.04%). EoE has also been considered a late manifestation of the allergic march, with an increased risk of development if preceded by any atopic condition, particularly by IgE food allergy [75, 76].

In some individuals with respiratory allergy, seasonal aeroallergens can exacerbate EoE [77, 78]. In 3% of EoE patients, a seasonal variation of esophageal eosinophilia was observed, and in 14% of cases, aeroallergens were considered triggers [79]. EoE may also develop after oral or, more rarely, sublingual allergen immunotherapy, as shown by two systematic reviews and a meta-analysis. EoE was detected as a complication of oral immunotherapy for various foods, such as milk, egg, and peanuts, in 2.7% to 5.7% of cases, with usual improvement of clinical and histological manifestations after discontinuation of immunotherapy. However, the real prevalence of EoE in patients who underwent immunotherapy is still uncertain, as the vast majority of patients did not undergo endoscopy [80].

Nonetheless, different phenotypes and endotypes exist in EoE [81], and many patients with EoE do not have atopic comorbidity, Th2 markers of inflammation, or elevated IgE, particularly in cases of narrow-caliber esophagus or fibrotic findings [82]. However, it has been proposed that the more severe forms of EoE may represent the natural evolution of untreated Th2 inflammation [82].

Q 4.3: Is there a higher prevalence of other gastrointestinal immune-mediated disorders, such as celiac disease, inflammatory bowel disease, or other eosinophilic gastrointestinal disorders, in children with EoE compared to the general population?

Statement 4.3

An unclear association exists between EoE and other gastrointestinal immune-mediated disorders, such as celiac disease and inflammatory bowel disease (IBD), despite reports suggesting an increased prevalence of these conditions in EoE patients.

[low quality of evidence]

[Vote result: Strongly agree: 48%; agree: 52%, neutral: 0%, disagree: 0%, strongly disagree: 0%]

Recommendation 4.3

During the initial endoscopic evaluation, we suggest obtaining multiple biopsies from the esophagus, stomach, and duodenum to screen for potential concomitant gastrointestinal disorders such as celiac disease, IBD, and eosinophilic gastroenteritis.

[low quality of evidence, grade of recommendation: weak]

[Vote result: Strongly agree: 94%; agree: 6%, neutral: 0%, disagree: 0%, strongly disagree: 0%]

Practice Point:

Consider performing endoscopy in patients with a known diagnosis of gastrointestinal disorders (e.g., celiac disease, inflammatory bowel disease) who develop new symptoms of esophageal dysfunction, to assess for potential EoE or other esophageal pathology.

Summary of evidence

EoE, celiac disease (CeD), and IBD are immune-mediated diseases of the gastrointestinal tract that share the risk of concurrent autoimmunity. There is an increased prevalence of CeD and IBD among patients with EoE, but the association is not always confirmed, and it is unclear whether it is causal or accidental [72]. Studies have reported a range of prevalence of CeD in EoE, from 0.16% to 57.1%, and a range of prevalence of EoE in CeD, from 0% to 10.7% [83, 84]. However, some studies may be biased towards positive associations, and there are concerns about the correct definition of EoE in children with CeD. Nonetheless, some studies have found an association between EoE and CeD even with strict definitions of EoE.

The prevalence of CeD and IBD among patients with EoE is higher than in the general population, but it is not clear whether the association is causal or accidental [83]. Studies have shown that the frequency of HLA DQ2 and DQ8 is similar between patients with EoE and healthy individuals, and there is no difference in esophageal eosinophilia infiltrate in children with CeD and in those without CeD [85]. Thus, celiac disease could be sought in selected children who have additional symptoms with respect to esophageal dysfunction [86]. Additionally, the prevalence of EoE among IBD patients and of IBD among EoE patients is higher than EoE and IBD alone in the general population [87, 88].

EoE and IBD have a higher prevalence when occurring together than when they occur alone, both in adults and in children. However, the relationship between EoE and IBD is not fully confirmed. IBD appears to be protective against developing EoE when treated with anti-tumor necrosis factor therapy. In contrast, an inverse relationship was found between EoE and Crohn’s disease but not with ulcerative colitis [89]. In the absence of symptoms or suggestive signs, diagnostic tests for IBD are not indicated in children with EoE and vice versa.

Q 4.4: Is there an increased risk of EoE in children with Esophageal Atresia (EA) compared to the general population?

Statement 4.4

Esophageal atresia patients may have a higher risk of developing EoE and should be screened with endoscopy and biopsies, as EoE in this population represents a specific subtype and requires tailored treatment strategies.

[moderate quality of evidence]

[Vote result: Strongly agree: 88%; agree: 12%, neutral: 0%, disagree: 0%, strongly disagree: 0%]

Recommendation 4.4

We suggest considering EoE in children with esophageal atresia who have persistent symptoms despite standard antireflux treatment, worsening dysphagia, and recurrent strictures.

[low quality of evidence, grade of recommendation: weak][low quality of evidence, grade of recommendation: weak]

[Vote result: Strongly agree: 78%; agree: 22%, neutral: 0%, disagree: 0%, strongly disagree: 0%]

Summary of evidence.

EoE is prevalent in children with EA, with some studies reporting a higher prevalence ranging from 9.5% to 30% [90–92]. Adolescents with EA have a high prevalence of EoE due to dysregulated baseline epithelial barrier and type 2-associated genes [93]. EoE should be excluded in EA patients presenting with certain symptoms and biopsies should be obtained even in asymptomatic patients during endoscopy [92]. While routine biopsies in all patients may cause underestimation of EoE, eosinophilia may also be related to EA complications rather than EoE, leading to overdiagnosis. Tailored treatment strategies are necessary for EoE in EA according to different EoE phenotypes [94].

Q 4.5: Is there an increased risk of psychiatric conditions in children with EoE?

Statement 4.5

Psychiatric comorbidities are common in children with EoE and should be considered when assessing symptoms.

Recommendation 4.4

We recommend considering referral to child psychiatry services for EoE patients exhibiting symptoms or signs of psychiatric conditions and ensuring psychological support is integrated into their disease management.

[low quality of evidence, grade of recommendation: weak]

[Vote result: Strongly agree: 84%; agree: 16%, neutral: 0%, disagree: 0%, strongly disagree: 0%]

Summary of evidence

A review of medical records in 950 adult EoE patients found that 31% had at least one psychiatric or neuropsychiatric condition, with depression (12%) and anxiety (9.3%) being the most common [95]. Another study of 705 EoE patients reported depression and anxiety in 15.5% of those under 17 [96]. The rates increased with age, with 24% of adults over 18 affected, compared to 9.3% of children [97]. A recent study also found that symptom-specific anxiety and hypervigilance, measured by the Esophageal Hypervigilance and Anxiety Scale (EHAS), were the main predictors of worsened dysphagia symptoms, regardless of endoscopic or histologic severity [98]. Therefore, referring EoE patients to mental health services should be part of clinical care when needed.

### Endoscopy

Q 5.1: Which endoscopic findings are important for diagnosing and monitoring EoE?

Statement 5.1

Endoscopic findings—including edema, furrows, white spots, rings, strictures, and trachealization—are valuable in the diagnostic process and in distinguishing between the inflammatory phenotype (edema, furrows, white spots) and the fibrotic phenotype (rings, strictures, trachealization) of EoE. However, these findings alone are not sufficient for diagnosis and should be correlated with histological and clinical assessment.

[high quality of evidence]

[Vote result: Strongly agree: 68%; agree: 32%, neutral: 0%, disagree: 0%, strongly disagree: 0%]

Practice Point:

Consider using the EoE Endoscopic Reference Score (EREFS) to standardize patient assessment and differentiate between inflammatory and fibrotic phenotypic manifestations of EoE in both clinical practice and research settings.

Summary of evidence

Eosinophilic esophagitis (EoE) can be identified by endoscopic findings such as esophageal pallor, linear grooves, white plaques, rings, and reduced caliber esophagus. However, 20–25% of EoE cases can have a normal-appearing esophagus [99]. The diagnosis of EoE is based on an eosinophilic infiltrate ≥ 15 eosinophils in at least one microscopy high-power field, and endoscopic scores can be used to grade the severity of lesions [100]. The Endoscopic Reference Score (EREFS) includes edema, rings, exudates, furrows, and strictures as major features of EoE [101]. Endoscopic findings vary with age; younger patients tend to have white plaques and normal-appearing esophagus, while adults tend to have strictures, narrow-caliber esophagus, rings, and crepe-paper mucosa [2]. Differentiating between inflammatory and fibrostenotic features of EoE is possible through endoscopic evaluation. Therefore, the use of EREFS in conjunction with symptoms and histology is encouraged to standardize patient assessment and differentiate the phenotypic manifestation of EoE in both clinical practice and research settings [101].

Q 5.2: What is the recommended biopsy protocol for the diagnosis and monitoring of EoE?

Statement 5.2

For an accurate diagnosis of EoE, multiple esophageal biopsies should be obtained from areas with endoscopic mucosal abnormalities to enhance diagnostic yield.

[high quality of evidence]

[Vote result: Strongly agree: 48%; agree: 52%, neutral: 0%, disagree: 0%, strongly disagree: 0%]

Recommendation 5.2:

We recommend obtaining at least six esophageal biopsies (two each from the proximal, mid, and distal esophagus) during the initial evaluation of suspected EoE, with a focus on areas of endoscopic mucosal abnormalities. For follow-up assessments, at least four biopsies should be taken to evaluate treatment response and ensure disease control.

[high quality of evidence, grade of recommendation: strong]

[Vote result: Strongly agree: 98%; agree: 2%, neutral: 0%, disagree: 0%, strongly disagree: 0%]

Practice Points:

- *Esophageal biopsies should be obtained even if the mucosa appears normal, as histologic abnormalities may still be present.*
- *During an episode of food bolus impaction, esophageal biopsies should always be performed to assess for underlying EoE or other esophageal pathology.*

Summary of evidence

The distribution of intraepithelial eosinophil infiltration in EoE is patchy, requiring multiple biopsies to increase diagnostic accuracy [102, 103]. Studies have shown that taking at least 6 to 9 esophageal mucosal samples increases sensitivity to 100% [104, 105]. Biopsy sampling should focus on areas with endoscopic mucosal abnormalities, such as white exudates and longitudinal furrows, associated with higher peak eosinophil counts [105]. In children with EoE, biopsies should be randomly taken from the proximal and distal esophagus, irrespective of endoscopic mucosal appearance, as normal mucosa is commonly seen [104]. Histologic examination of only the mid esophagus offers little diagnostic value in pediatric patients. Gastric and duodenal biopsies should be obtained initially to exclude eosinophilic gastroenteritis but are not strictly necessary during follow-up. PPI treatment can alter endoscopic and histologic findings, so PPIs should be discontinued for three to four weeks before endoscopy in patients with suspected EoE [40].

Q 5.3: How safe and effective is endoscopic dilation as a treatment option for EoE?

Statement 5.3

Endoscopic dilation is a safe and effective treatment for EoE patients with esophageal strictures and dysphagia who have persistent symptoms despite medical and/or dietary therapy.

[high quality of evidence]

[Vote result: Strongly agree: 75%; agree: 20%, neutral: 5%, disagree: 0%, strongly disagree: 0%]

Recommendation 5.3:

We recommend considering a short course of systemic steroids as an alternative to dilation in cases of moderate to severe esophageal strictures with an inflammatory component.

[low quality of evidence, grade of recommendation: weak]

[Vote result: Strongly agree: 72%; agree: 20%, neutral: 8%, disagree: 0%, strongly disagree: 0%]

Practice Point:

If a patient with EoE has persistent dysphagia despite adequate treatment and histological remission, a barium esophagram may be considered to identify strictures that may not be visible on upper endoscopy.

Summary of evidence

Esophageal strictures are among the most severe complications of EoE, resulting from longstanding eosinophilic inflammation. While uncommon in pediatric patients due to the shorter disease duration, they can still occur [2]. The diagnosis of esophageal strictures relies primarily on endoscopic assessment of esophageal caliber. However, endoscopy may underestimate strictures compared to barium esophagram, and both techniques should be used for a comprehensive evaluation in selected patients [106]. Esophageal dilation with through-the-scope balloons or bougies has been the treatment of choice for other esophageal strictures, with symptomatic improvement observed in 95% of patients, regardless of the dilation method used [107]. Although long-term efficacy data are limited, inflammation control remains crucial in managing fibrostenotic EoE, as it reduces the need for repeated dilations [108].

Contrary to early concerns, recent studies have shown that complication rates for dilation in EoE are comparable to those for other esophageal conditions: perforation: 0.38%–0.61%; hemorrhage: 0.05%–0.38%; post-procedure hospitalization: 0.6%–0.74% [108]. While many patients experience transient thoracic discomfort, no mortality has been reported.

Most mild EoE-associated inflammatory strictures in children respond well to standard treatments such as PPIs, topical steroids, or dietary therapy. A study in adults found that dilation for mild strictures did not provide additional symptom relief compared to standard treatment alone [109]. In pediatric patients, shorter disease duration suggests that inflammatory strictures are more common than fibrostenotic ones, explaining the high response rate to short courses of systemic steroids [110].

However, in cases of severe dysphagia due to significant strictures, systemic steroids alone may not be sufficient, and dilation may be required. A retrospective study of 20 children with moderate to severe strictures found that a short course of systemic steroids (2–4 weeks followed by tapering) resulted in symptomatic improvement in all patients, with complete endoscopic resolution in 95%, and only three patients requiring dilation [110]. This contrasts with primary dilation treatment, where most patients required at least one additional dilation.

Dilation is recommended for children with persistent dysphagia despite histological improvement with standard treatment, as mild fibrostenotic changes may impair motility and lead to ongoing dysphagia or food bolus impaction. However, dilation alone does not address underlying inflammation and should be used alongside standard induction and maintenance therapy.

Q5.4: Is endoscopic follow up suggested for monitoring EoE?

Statement 5.4

Endoscopy with biopsies is a crucial diagnostic and monitoring tool for assessing therapy response and evaluating disease complications in patients with EoE.

[moderate quality of evidence]

[Vote result: Strongly agree: 85%; agree: 15%, neutral: 0%, disagree: 0%, strongly disagree: 0%]

Recommendation 5.4

We recommend repeating endoscopy with biopsy 8–12 weeks after initiating therapy and after any treatment modifications to assess response in patients with EoE.

[high quality of evidence, grade of recommendation: strong]

[Vote result: Strongly agree: 82%; agree: 12%, neutral: 6%, disagree: 0%, strongly disagree: 0%]

Practice Points

- *A suggested follow-up schedule for endoscopy during the maintenance phase in EoE patients could be every 1–2 years, or more frequently in those with persistent symptoms or inadequate treatment response.*
- *The follow-up schedule should be individualized, considering the patient’s response to therapy and the presence of symptoms.*

Summary of evidence

The goal of EoE therapy is to relieve symptoms, improve endoscopic and histologic findings, and prevent disease progression [111]. While there is no fixed schedule for endoscopic monitoring, it should be individualized based on therapy response. Mucosal healing and eosinophilic infiltration should be assessed after therapy modifications (e.g., steroids or diet changes) or symptom relapse. Endoscopic evaluation should include gross lesion assessment (exudates, furrows, edema, rings), and biopsies should be routinely performed, as clinical symptoms do not always correlate with histologic severity.

There is limited data on optimal follow-up intervals for patients on maintenance therapy [112]. A 7.2-year study in adults found that untreated EoE led to persistent dysphagia, inflammation, and strictures, while a pediatric study confirmed the chronic, relapsing nature of EoE, even with steroid treatment [113]. Some patients lose long-term response to therapy, necessitating regular follow-up, including endoscopy and biopsies [114].

Importantly, asymptomatic patients may still develop fibrosis and strictures, reinforcing the need for routine follow-up. A study of 159 EoE patients on steroids found fewer strictures in those with closer follow-up [115]. For relapsing patients, endoscopy with biopsies is recommended, even if they remain on treatment. For those in remission, consistent follow-up is crucial, as gaps in care have been linked to increased disease activity and complications [116]. Patients in clinical and histologic remission should undergo clinical assessment 12–24 months after their last endoscopy, with further endoscopies based on individual risk of recurrence [112].

### Histology

Q 6.1: How is the eosinophil mucosal density threshold defined for the diagnosis of EoE?

Statement 6.1

A peak eosinophil count ≥ 15 eos/hpf (about 60 eos/mm2) in one or more esophageal biopsies is considered diagnostic of EoE.

[moderate quality of evidence]

[Vote result: Strongly agree: 56%; agree: 44%, neutral: 0%, disagree: 0%, strongly disagree: 0%]

Summary of evidence:

The adoption of a cut-off of ≥ 15 eos/hpf has reduced diagnostic variability and is now commonly used in clinical practice [117]. Studies have demonstrated that this threshold provides 100% sensitivity and 96% specificity for diagnosing EoE. [118]

However, eosinophil counts can vary due to differences in microscope hpf areas [119]. Measuring eosinophil density in eos/mm [2] may offer greater reliability than eos/hpf and can be reported alongside eosinophil counts. Additionally, communication with pathologists is recommended in cases of uncertain findings [117].

Counting eosinophils can be challenging when they are degranulated, clustered in micro-abscesses, or embedded in parakeratotic crusts [120]. When eosinophil levels are mildly elevated (< 20/hpf), pathologists should report the highest count per hpf and provide ranges of mucosal eosinophilia when numbers are higher (e.g., 15–50, > 50, or > 100 per hpf) [120].

Since the 15 eos/hpf threshold is arbitrary, evaluating additional histologic features and clinical context is crucial, especially in cases with borderline eosinophil counts. This approach is strongly recommended in research settings to ensure diagnostic accuracy.

Q 6.2: How can the histological assessment of EoE be expanded beyond peak eosinophil counts to include additional markers?

Statement 6.2

Beyond peak eosinophil counts, the histological assessment of EoE can be expanded by evaluating additional histopathologic features, including eosinophil degranulation, basal zone hyperplasia, spongiosis, lamina propria fibrosis, and dyskeratosis.

[low quality of evidence]

[Vote result: Strongly agree: 48%; agree: 52%, neutral: 0%, disagree: 0%, strongly disagree: 0%]

Recommendation 6.2

Communication with pathologists and the assessment of additional clinical and histological features are recommended to interpret borderline peak eosinophil counts and enhance diagnostic accuracy in EoE.

[low quality of evidence, grade of recommendation: strong]

[Vote result: Strongly agree: 84%; agree: 16%, neutral: 0%, disagree: 0%, strongly disagree: 0%]

Practice Point

The EoE Histology Scoring System (EoEHSS) can be used as an objective tool to assess disease activity and severity in EoE histological samples, providing a standardized evaluation beyond peak eosinophil counts.

Summary of evidence:

The peak eosinophil count ≥ 15 intraepithelial eos/hpf, while a key diagnostic feature, may have limitations, particularly in borderline cases. Additional histological features, such as eosinophil abscesses, basal zone hyperplasia, dilated intercellular spaces, eosinophil surface layering, papillary elongation of the squamous epithelium, and thickened lamina propria fibers, can also be considered [121]. Although not specific to EoE, these features are typically more common and severe in EoE patients.

Assessing the severity and extent of inflammation may provide further diagnostic value. A validated histological scoring system, the EoE Histology Scoring System (EoEHSS), classifies histologic abnormalities based on severity and extent using a four-point scale across eight EoE-associated features [122]. A derived score, the EoE Histology Remission Score (EoEHRS), has been proposed, demonstrating strong associations with reduced disease activity and lower symptom scores [123, 124].

The EoEHSS is a reliable and efficient tool for evaluating multiple histological abnormalities in EoE biopsies. It requires minimal pathologist training, takes less than a minute per biopsy slide, and is more strongly associated with treatment status than the peak eosinophil count alone [125].

Q 6.3: How can histological features be used to distinguish eosinophilic esophagitis from gastroesophageal disease?

Statement 6.3

The relationship between EoE and GERD is complex, as they can share similar histological features and coexist in the same patient. However, clinical and endoscopic findings can assist in differentiating the two conditions. Additionally, their coexistence may result from distinct pathogenetic mechanisms.

[moderate quality of evidence]

[Vote result: Strongly agree: 65%; agree: 20%, neutral: 15%, disagree: 0%, strongly disagree: 0%]

Practice Point:

The presence of marked basal zone hyperplasia, elongated vascular papillae, and eosinophils in the proximal esophageal mucosa is more suggestive of EoE rather than GERD.

Summary of evidence

GERD is typically associated with eosinophil counts below 5 eos/hpf. However, distinguishing GERD from EoE can be challenging, as they share similar histological features and may coexist in the same patient [12, 126]. Additionally, eosinophils are a feature of acid-induced esophagitis, particularly in children, and severe GERD can result in eosinophil counts exceeding 15 eos/hpf [117].

In EoE, marked basal zone hyperplasia and elongated vascular papillae are more commonly observed [127]. While clinical and endoscopic findings can aid in differentiating EoE from GERD, their relationship is complex, as they may coexist due to different pathogenetic mechanisms [128].EoE may induce secondary reflux due to decreased esophageal compliance or dysmotility.GERD may compromise epithelial barrier integrity, leading to antigen exposure and subsequent eosinophilia [16]. This interplay underscores the need for a comprehensive diagnostic approach that integrates clinical, endoscopic, and histologic findings.

Q 6.4: How is histological remission defined in eosinophilic esophagitis?

Statement 6.4

Histological remission in eosinophilic esophagitis is defined as a peak eosinophil count of ≤ 15 eosinophils/hpf.

[moderate quality of evidence]

[Vote result: Strongly agree: 88%; agree: 12%, neutral: 0%, disagree: 0%, strongly disagree: 0%]

Summary of evidence:

The goal of therapy in clinical care and research is to reduce eosinophil density below the diagnostic threshold (< 15 eos/hpf) [111]. While a more stringent criterion of ≤ 5 eos/hpf has been proposed in numerous interventional trials to maximize symptomatic and endoscopic responses, achieving this level in routine clinical practice can be challenging. [129]

To further corroborate histological remission, additional assessment of other histologic markers of eosinophilic inflammation—such as eosinophilic microabscesses, eosinophil surface layering, extracellular eosinophil granules, basal cell hyperplasia, and dilated intercellular spaces—may provide valuable insights [129, 130].

Additionally, achieving deep remission with the complete resolution of histological abnormalities could be considered an optimal therapeutic goal.

### Motility assessment

Q7.1: What is the role of esophageal pH-impedance monitoring in pediatric EoE diagnosis and follow-up?

Statement 7.1

The available evidence on the role of esophageal pH-impedance in the diagnostic workup and in the follow-up of pediatric EoE is limited.

[low quality of evidence]

[Vote result: Strongly agree: 73%; agree: 13%, neutral: 14%, disagree: 0%, strongly disagree: 0%]

Practice Point

MII-pH might be considered in selected cases to define reflux related disorders.

Summary of evidence

Esophageal pH-impedance monitoring (MII-pH) is currently not included in the diagnostic work-up of EoE. However, recent studies on adult populations suggested its possible role in both EoE diagnosis and follow-up [131]. It has been demonstrated that esophageal baseline impedance assessed through MII-pH may be a marker of esophageal epithelial integrity in GERD and also in EoE patients [132, 133]. Indeed, low esophageal impedance baseline are typically associated with active EoE and they correlate to maximum eosinophil counts greater than 15 eos/HPF [133]. Moreover, mucosal permeability abnormalities as assessed by MII-pH in patients with EoE can be either uniform or patchy, but tend to be present in almost all patients with EoE regardless of histologic activity defined by the current criterion standard of maximum eos/HPF [134].

A further intriguing finding is that EoE patients may have a specific MII pattern. Of note, esophageal impedance values were found consistently low throughout the esophagus differently from patients with GERD, which have low mucosal impedance closer to the squamocolumnar junction and increased values axially along the esophagus [135]. Recent studies have confirmed these findings, showing impaired esophageal mucosal integrity in patients with EoE and suggesting that increased epithelial permeability has a possible role in the presentation of allergens to the immune system [135].

In conclusion, there is some scientific evidence that esophageal impedance patterns may identify patients with EoE vs normal mucosa or GERD with high levels of sensitivity. Recently, values of baseline impedance have been testes also as a non-invasive method to evaluate disease activity. Indeed, patients with active eosinophilic inflammation exhibit low levels of baseline, but future studies are needed to assess whether it can be used in routine clinical practice [136].

Q7.2: What is the value of esophageal manometry in EoE diagnosis and follow-up in children?

Recommendation 7.2

We suggest not using esophageal manometry for the diagnosis and during the routine follow-up of EoE in children.

[low quality of evidence, grade of recommendation: weak]

Recommendation 7.3

We suggest considering using esophageal manometry only to rule out achalasia and other major obstructive motor disorders in EoE patients with persistent symptoms after adequate treatment.

[low quality of evidence, grade of recommendation: weak]

[Vote result: Strongly agree: 68%; agree: 16%, neutral: 16%, disagree: 0%, strongly disagree: 0%]

Summary of evidence

Abnormalities in esophageal motility and distensibility are considered the most common causes of dysphagia and food impaction, particularly when obstructive abnormalities, such as esophageal strictures, are not detected [137]. Several mechanisms have been implicated in the pathogenesis of esophageal dysmotility, but its exact etiology remains unclear.

Although esophageal manometry, especially high-resolution manometry (HRM), allows for an accurate characterization of esophageal motor function, only a few studies have explored its role in EoE, with most conducted in adults [138]. Using the Chicago classification system, esophageal motor abnormalities have been identified in 20–80% of EoE patients [69]. However, no pathognomonic motility pattern has been established due to the high heterogeneity of results.

Several motility disturbances have been reported in EoE patients, including: weak or failed peristalsis; panesophageal pressurization; elevated distal intrabolus pressure (especially in fibrostenotic disease), with or without features of esophagogastric junction outflow obstruction; hypertensive peristalsis and jackhammer esophagus [139]. Motor abnormalities on HRM do not appear to correlate with the severity of dysphagia or endoscopic findings [140]. However, they are reported to be more prevalent in EoE patients with prolonged disease [141] and tend to improve after treatment with topical steroids. [142].

Q7.3: What is the role of EndoFLIP in the management of EoE patients?

Statement 7.3

EndoFLIP might represent a useful tool to assess esophageal remodeling in EoE.

[low quality of evidence]

[Vote result: Strongly agree: 73%; agree: 17%, neutral: 0%, disagree: 0%, strongly disagree: 0%]

Practice point

Consider Endoflip in pediatric EoE patients with persistent symptoms after adequate treatment.

Summary of evidence:

A new endoluminal functional lumen imaging probe, the EndoFLIP system, allows the evaluation of the effects of EoE on the esophageal wall, particularly distensibility and esophageal luminal diameter [143]. A preliminary study has initially demonstrated a significant reduction in esophageal distensibility in EoE patients, since an increase in balloon volume and pressure does not result in a corresponding increase in cross-sectional area, suggesting fibrosis and a fixed lumen [143]. This phenomenon is termed “the distensibility plateau”. Diffuse esophageal narrowing correlates with lower distensibility plateau values, which in turn correlate with lamina propria fibrosis and increased episodes of food impaction [144]. Impaired distensibility is associated with age and disease duration [145]. Moreover, it can be partially reversible in case of successful medical treatment (steroids or diet); in contrast, distensibility does not change in case of treatment failure [146]. Thus, in the setting of persisting symptoms despite medical, dietary or endoscopic treatment, measurements of distensibility and luminal diameter using EndoFLIP could provide evidence for treatment escalation [143]. Similarly, in the presence of histologic remission with persisting symptoms, EndoFLIP may suggest that symptoms could be due to increased esophageal stiffness or reduced distensibility because of chronic esophageal fibrosis, which might indicate the need for esophageal dilation. Because of this type of assessment, EndoFLIP distensibility measurements have been recently considered as secondary endpoints in EoE clinical trials with topical steroids and biologics. However, the role of EndoFLIP in routine EoE clinical care is not yet well established, and further prospective studies are required. A recent expert consensus on how to interpret its results in practice has been published [147].

### Therapy

Q8.1: What should the therapeutic goals be? Resolution of symptoms, resolution of inflammation, or both?

Recommendation 8.1

The goal of EoE treatment includes improvements in all disease domains: symptoms, endoscopy and histology.

[moderate quality of evidence, grade of recommendation: strong]

[Vote result: Strongly agree: 98%; agree: 2%, neutral: 0%, disagree: 0%, strongly disagree: 0%]

Practice Point

Therapies should be adjusted or modified if the disease is not controlled in one or more disease domains (symptoms, endoscopy, and histology).

Summary of evidence

The main goals of EoE therapy are to eliminate symptoms, reduce mucosal inflammation, resolve disease consequences, and prevent complications [111]. However, no standardized definition of treatment response currently exists [39].

For regulatory trials, the FDA recommends co-primary endpoints of histopathology (peak eosinophil density) and symptom-based patient-reported outcomes [148]. Endoscopic activity is often a secondary endpoint to assess treatment efficacy [111]. While endoscopy can detect esophageal remodeling (e.g., rings and strictures), barium esophagram and impedance planimetry (FLIP) may be more accurate for identifying and quantifying remodeling [147].

An effective treatment should improve symptoms, reduce histologic eosinophilia, and improve endoscopic features. Thresholds for response are generally considered as: 1) resolution of dysphagia without diet modification; 2) fewer than 15 eos/hpf in the esophageal epithelium; 3) reduction in inflammatory endoscopic features (exudates, furrows, edema) with esophageal diameter ≥ 16 mm.

A complete responder should meet all three criteria, while a definite non-responder would fail to improve in any. Partial responses with discordant improvements pose a management challenge for physicians [149].

Q8.2: Is PPI therapy effective and safe for inducing and maintaining remission in EoE?

Statement 8.2.1

PPI therapy is safe and effective in inducing remission in pediatric patients with EoE.

[moderate quality of evidence]

[Vote result: Strongly agree: 48%; agree: 52%, neutral: 0%, disagree: 0%, strongly disagree: 0%]

Statement 8.2.2

PPI treatment can maintain clinical and histological remission in patients with EoE who respond to this therapy.

[low quality of evidence]

[Vote result: Strongly agree: 48%; agree: 52%, neutral: 0%, disagree: 0%, strongly disagree: 0%]

Recommendation 8.2.1

We recommend a PPI therapy of 2 mg/kg/day to induce remission in EoE children for a period of 8–12 weeks.

[moderate quality of evidence, grade of recommendation: strong]

[Vote result: Strongly agree: 86%; agree: 10%, neutral: 4%, disagree: 0%, strongly disagree: 0%]

Recommendation 8.2.2

We suggest reducing PPI therapy to 1 mg/kg/day to maintain remission in EoE children.

[low quality of evidence, grade of recommendation: weak]

[Vote result: Strongly agree: 66%; agree: 20%, neutral: 14%, disagree: 0%, strongly disagree: 0%]

Practice Point

Older children with minimal or no endoscopic lesions are more likely to respond to PPI therapy.

Maintenance therapy should be continued for at least 1 year, as the risk of recurrence is higher after treatment interruption.

Summary of evidence

Several randomized controlled trials and meta-analyses support PPI therapy as an effective first-line treatment for EoE, with histologic remission rates ranging from 33 to 57% and symptomatic improvement in approximately 60% [150, 151]. A meta-analysis of 33 studies involving 619 patients reported PPI-induced histologic remission in 50.5% of cases, with no significant differences based on age, study design, or type of PPI used [152]. The therapeutic effects of PPIs are attributed to their ability to reduce acid exposure and suppress eotaxin-3, a Th2 cytokine involved in eosinophil recruitment [151]. Higher remission rates of 70–80% have been observed in patients with GERD, although esophageal pH monitoring has not consistently predicted treatment response. Additionally, a negative correlation exists between eosinophil count and PPI response, but no clear threshold for responsiveness has been established. Older, non-atopic children with mild or no endoscopic lesions appear to have higher response rates [2].

Data on the long-term maintenance of remission with PPIs in pediatric EoE is limited. However, a study of 57 children found that after initial high-dose esomeprazole therapy, 86% remained symptom-free, and 70.1% maintained histologic remission after one year [153]. Step-down strategies sustained remission in 68.5% to 85.3% of children over 7 to 16 months [154]. Similar responses have been observed in adults on lower PPI doses [155]. Loss of response was more common in CYP2C19 rapid metabolizers, while children with STAT6 variants had a higher risk of relapse after one year [156].

Although PPIs are generally safe, common side effects include headaches and diarrhea, and concerns have been raised about microbiota disruption, impaired nutrient absorption, and increased risks of food allergies, asthma, and bone fractures [157, 158]. Early PPI exposure has been associated with a higher risk of developing EoE, with an odds ratio of 6.05 [159]. Given these potential risks, the long-term safety of PPIs should be carefully considered. Despite these concerns, PPIs remain a viable first-line treatment for EoE, typically administered as omeprazole 2 mg/kg twice daily for 8–12 weeks to assess response, with careful monitoring for long-term use [150, 160].

Q 8.3: Are topical steroids effective in inducing and maintaining remission of EoE?

Statement 8.3.1

Topical steroids are safe and effective in inducing clinical and histological remission in pediatric patients with EoE.

[high quality of evidence]

[Vote result: Strongly agree: 95%; agree: 5%, neutral: 0%, disagree: 0%, strongly disagree: 0%]

Statement 8.3.2

In steroids responsive children, topical steroids are safe and effective in maintaining remission.

[moderate quality of evidence].

[Vote result: Strongly agree: 46%; agree: 54%, neutral: 0%, disagree: 0%, strongly disagree: 0%]

Recommendation 8.3

We suggest reducing swallowed topical steroids to maintain remission in children with EoE once complete remission has been achieved.

[moderate quality of evidence, grade of recommendation: strong]

[Vote result: Strongly agree: 86%; agree: 10%, neutral: 4%, disagree: 0%, strongly disagree: 0%]

Practice Points

Budesonide and fluticasone can be both used in children with variable dosages according to the type of formulation used. The suggested induction doses in children are 880–1760 mcg/day for fluticasone propionate and 1–2 mg/day for viscous budesonide depending on height (< or > 150 cm) and/or age (< or > 10 years).

During the maintenance phase, budesonide and fluticasone dosage might be lowered if patients are able to remain in symptomatic, endoscopic, and histological remission.

Maintenance therapy should be continued for at least 1 year, as the risk of recurrence is higher after treatment interruption.

A gradual dose reduction may be considered to identify the lowest effective dose and minimize potential long-term adverse events.

Summary of evidence

Numerous randomized controlled trials, summarized in systematic reviews and meta-analyses, support the efficacy of topical steroids in achieving clinical and histological remission in EoE patients [150, 161, 162]. In pediatric populations, available options include nebulized fluticasone propionate and viscous budesonide. Fluticasone is typically delivered via an inhalation device without a spacer, while budesonide is administered as a viscous solution [163]. Induction doses for children range from 880–1760 mcg/day for fluticasone and 1–2 mg/day for budesonide. Since efficacy is linked to mucosal exposure time, doses should be split into morning and evening administrations, with caregivers advised to prevent eating, drinking, or tooth brushing for at least 30 min post-dose [164].

In 2017, the European Medicines Agency (EMA) approved an effervescent budesonide tablet for adults with EoE, though this formulation is not yet approved for children [165]. In pediatric studies, both fluticasone and budesonide have demonstrated significantly higher histologic remission rates compared to placebo, effectively reducing eosinophil counts below 15 cells/hpf [150, 166]. Additionally, topical steroids have shown superior symptom relief, particularly in dysphagia improvement [167]. While no direct pediatric trials have compared the efficacy of budesonide and fluticasone, a randomized placebo-controlled study in adults reported similar clinical and histological outcomes between the two treatments [163].

Swallowed steroids have been well tolerated, with no significant side effects during induction therapy, aside from asymptomatic oral or esophageal candidiasis, observed in 5–26% of patients [161]. Given the chronic nature of EoE, maintenance therapy is recommended to prevent relapses and disease progression to fibrosis [168]. Although data on long-term use are limited, guidelines suggest reducing the induction dose by 50% for maintenance therapy [7, 169]. Both fluticasone and budesonide have shown similar efficacy in maintaining remission, with sustained response rates of 73% at three months and over 59% at two years for fluticasone-treated children [169, 170].

Long-term safety data on topical steroids remain inconclusive but are generally reassuring [171]. Beyond candidiasis, adrenal suppression has been reported in 10% of children receiving high-dose fluticasone for more than six months, though no clinical signs of adrenal insufficiency or growth retardation have been observed [171, 172]. Cortisol monitoring is recommended in children on prolonged high-dose therapy [173, 174]. A recent strategy of dose reduction to the lowest effective level has shown promise in maintaining remission for up to 84 weeks [175]. Endoscopic and histological evaluations are necessary to confirm treatment effectiveness following dose adjustments, with the goal of minimizing long-term adverse effects [175].

Q 8.4: What is the efficacy of immunomodulators in treating EoE?

Statement 8.4

Evidence for use of immunodulators in EoE is small.

[low quality of evidence]

[Vote result: Strongly agree: 62%; agree: 38%, neutral: 0%, disagree: 0%, strongly disagree: 0%]

Recommendation 8.4

We recommend not using immunodulators for the management of EoE in children due to limited evidence.

[low quality of evidence, grade of recommendation: strong]

Summary of evidence:

Since corticosteroids do not induce long-lasting remission in EoE, immunomodulators have been explored as a potential maintenance therapy. Retrospective reports on the use of azathioprine and 6-mercaptopurine in EoE are limited to a small series of four patients, all of whom had refractory disease. While treatment with immunomodulators led to normalized tissue eosinophilia, eosinophilic inflammation recurred after discontinuation [176]. However, the lack of control subjects and the presence of multiple confounding factors make it difficult to determine the true efficacy of immunomodulatory therapy in EoE.

Q 8.5: What is the efficacy of Anti-allergic drugs in EoE?

Statement 8.5

There is limited evidence regarding the use of montelukast, a leukotriene receptor antagonist, in patients with EoE**,** whereas the first-generation chemoattractant receptor-homologous molecule on Th2 cells (CRTH2) antagonist has shown in a phase 2 study modest clinic and histologic improvement in EoE.

[low quality of evidence]

[Vote result: Strongly agree: 48%; agree: 52%, neutral: 0%, disagree: 0%, strongly disagree: 0%]

Recommendation 8.5

We suggest not using cromolyn sodium or montelukast for the management of EoE in children due to limited evidence.

[low quality of evidence, grade of recommendation: strong]

Summary of evidence

Several antiallergic drugs used for rhinitis and asthma have shown little effect on EoE symptoms or esophageal inflammation. Cromolyn sodium has no proven benefit for EoE, as a small trial in 14 patients showed no clinical improvement after a 1-month course [177]. Despite its lack of significant side effects, it is not recommended for children with EoE. Similarly, there is insufficient evidence to support the use of leukotriene receptor antagonists like montelukast. Although one study reported clinical, but not histological, remission in 8 patients after 14 months of montelukast, most relapsed soon after stopping treatment [178]. A randomized trial found that montelukast did not significantly improve symptom remission compared to placebo at week 26, and another study showed it was ineffective in maintaining remission after a 6-month treatment [179, 180]. Thus, montelukast is not recommended as primary therapy for EoE [4].

A recent study investigated the CRTH2 antagonist OC000459 in 26 adults with EoE. This drug modestly reduced eosinophil load and physician assessments of disease activity but did not show substantial benefits compared to placebo [181].

Q 8.6: Is there a role for biologic drugs in the treatment of EoE?

Statement 8.6

Biologics targeting IL-4 and IL-13 have shown the most significant treatment benefits in both children and adolescents.

[high quality of evidence]

[Vote result: Strongly agree: 98%; agree: 2%, neutral: 0%, disagree: 0%, strongly disagree: 0%]

Recommendation 8.6

We recommend using approved biologics targeting IL-4 and IL-13 for the management of EoE in children after failure of conventional treatments.

[high quality of evidence, grade of recommendation: strong]

[Vote result: Strongly agree: 98%; agree: 2%, neutral: 0%, disagree: 0%, strongly disagree: 0%]

Practice Points

In selected cases of severe disease or multiple atopic conditions, dupilumab might be considered as an upfront therapy.

Weight-tiered dosing of dupilumab based on body weight should be considered: 200 mg every other week for patients ≥ 15 kg to < 30 kg, 300 mg every other week for patients ≥ 30 kg to < 40 kg, and 300 mg every week for patients ≥ 40 kg.

Summary of evidence

Biologics targeting molecules involved in the type-2 atopic cascade have been studied for EoE [182]. Dupilumab, an anti-IL-4 receptor-α antibody, showed positive results in a study of 47 adults with EoE, achieving histological remission in 82% of patients. It also improved endoscopic and histological scores, esophageal distensibility, and normalized type 2 inflammation-related gene expression [183]. In a Phase 3 trial, dupilumab led to histological remission in 60% and 59% of patients on weekly and biweekly doses, respectively, but only the weekly dose had significant clinical response [184]. Dupilumab is now approved for EoE in patients aged 12 years and older, with FDA and EMA approval recently extending to children aged 1–11. These data include severe patients refractory to other therapies [185]. While the role of Dupilumab as a first-line therapy still needs to be established, its ability to treat other atopic conditions and halt the Th2 inflammatory process suggests it could be considered as an upfront therapy in selected children [186].

IL-13 is another key cytokine in EoE pathogenesis. QAX576, an IL-13 antibody, decreased eosinophil numbers but had no effect on symptoms [187]. RPC4046 (cendakimab) reduced eosinophil counts and improved endoscopic, histological, and clinical measures in a phase 2 trial, with greater benefits at higher doses, suggesting potential to reduce fibrostenotic complications [188, 189]. Omalizumab, an anti-IgE antibody, failed to show significant effects in two studies [190, 191].

Anti-IL-5 therapies, reslizumab and mepolizumab, also showed mixed results. While reslizumab reduced esophageal eosinophil infiltration in children, histological remission was rare [192]. Mepolizumab, despite reducing eosinophil counts, did not improve symptoms or achieve remission in adults or children [193, 194]. Benralizumab, an anti-IL-5r alpha antibody, depleted eosinophils but did not improve symptoms, leading to the trial’s early termination [195]. These failures suggest non-eosinophil mediated inflammation plays a crucial role in EoE.

Vedolizumab, an anti-α4β7-integrin antibody, showed conflicting results in anecdotal cases, with some patients achieving histological remission and others seeing no benefit [196].

### Diet

Q 9.1: What is the role of the diet in the treatment of EoE?

Statement 9.1

Dietary therapy, along with PPIs and topical corticosteroids, is considered a first-line treatment for EoE.

[strong quality of evidence]

[Vote result: Strongly agree: 98%; agree: 2%, neutral: 0%, disagree: 0%, strongly disagree: 0%]

Summary of evidence

Food antigens are believed to be the primary triggers of EoE [197]. Unlike pharmacological treatments, which address the inflammatory effects of the disease, elimination diets directly target its underlying cause [198]. While extensive dietary restrictions can impact patients’ quality of life, dietary management remains a first-line therapy for EoE, alongside PPIs and topical corticosteroids [199].

Q 9.2: How to eliminate foods from the diet?

Statement 9.3

Given the comparable efficacy of less restrictive diets (one-food elimination diet) and more restrictive approaches (four- or six-food elimination diet), a step-up strategy is preferred, as it reduces the need for endoscopies, lowers costs, and improves patient compliance.

[high quality of evidence]

[Vote result: Strongly agree: 98%; agree: 2%, neutral: 0%, disagree: 0%, strongly disagree: 0%]

Recommendation 9.3

To accurately assess the effectiveness of dietary restriction therapy, the targeted food(s) should be eliminated for at least 4–8 weeks. At the end of this period, esophageal biopsies are necessary to determine histological remission, as symptoms and endoscopic findings alone are not sufficient. Remission is defined as fewer than 15 eosinophils per high-power field.

[high quality of evidence, grade of recommendation: strong]

[Vote result: Strongly agree: 98%; agree: 2%, neutral: 0%, disagree: 0%, strongly disagree: 0%]

Practice Point

Milk, wheat, egg, soy, fish/shellfish, and nuts are six major food groups implicated in the pathogenesis of EoE due to their allergenic potential. Given current evidence, it is now considered more practical to begin elimination therapy by first excluding milk as the primary potential trigger before removing additional foods if necessary.

Summary of evidence

The step-up approach is considered the most cost-effective strategy for an empiric elimination diet in EoE, as it reduces the number of endoscopies and shortens the food reintroduction period. First evaluated in 2018 in the largest multicenter diet study for EoE, this approach included 130 patients (25 children) from 14 centers [200]. All patients initially underwent a two-food elimination diet (TFED) excluding milk and wheat. Non-responders were then escalated to a four-food elimination diet (FFED) (adding soy and egg) and, if necessary, to a six-food elimination diet (SFED) (also excluding nuts and fish/shellfish). Histologic and symptomatic remission was achieved in 43% of patients after TFED, increasing to 60% after FFED and 79% with SFED as a rescue therapy [200].

More recently, two randomized controlled trials demonstrated comparable efficacy between a single-food elimination diet (excluding only cow’s milk) and more restrictive diets (four- and six-food elimination) [201, 202]. Based on these findings, it is now considered more practical to start dietary therapy by eliminating only milk as the initial potential trigger.

Q 9.5: Is dietary therapy able to maintain remission in the long-term period?

Statement 9.5

Eliminating identified trigger foods from the diet can effectively maintain remission in children with EoE.

[high quality of evidence]

[Vote result: Strongly agree: 48%; agree: 52%, neutral: 0%, disagree: 0%, strongly disagree: 0%]

Practice Point

Regular assessment of nutritional status is essential throughout the dietary treatment period to prevent malnutrition.

Summary of evidence

More recent studies have confirmed that adherence to dietary therapy sustains long-term histologic remission for up to five years, along with symptom resolution and improvement in endoscopic findings [203, 204]. Earlier research similarly demonstrated that patients on dietary therapy remained asymptomatic and maintained histological remission for up to three years [205].

### Practical suggestions for therapy selection, multidisciplinary management, and patient education

The choice of first-line therapy for EoE should be based on clinical presentation, endoscopic findings, histological profile, and patient preferences. In patients with mild disease, GERD-like symptoms, or adolescents, PPIs are preferred. TCS, administered via EoE-specific or galenic formulations, are recommended in cases with more pronounced inflammatory features on EREFS scoring. Dietary interventions, particularly a single-food elimination diet (e.g., cow’s milk), represent a valid alternative, with elemental diets reserved for the most severe or refractory presentations. In individuals showing fibrotic endoscopic features, severe disease, or significant concomitant atopic conditions, early initiation of biologic therapy such as dupilumab may be considered. As proposed in recent literature, EoE treatment can be conceptualized as a step-up pyramid, with PPIs, TCS, and diet at the base, and biologics positioned at the apex for refractory or severe phenotypes. However, a ‘top-down’ approach with early biologics might be justified in selected cases to prevent esophageal remodeling, particularly in pediatric patients or those with risk factors for disease progression [186]. Shared decision-making remains crucial to tailor therapy to disease severity, phenotype, and patient and family priorities, aiming to optimize long-term outcomes.

**Fig. 2:**
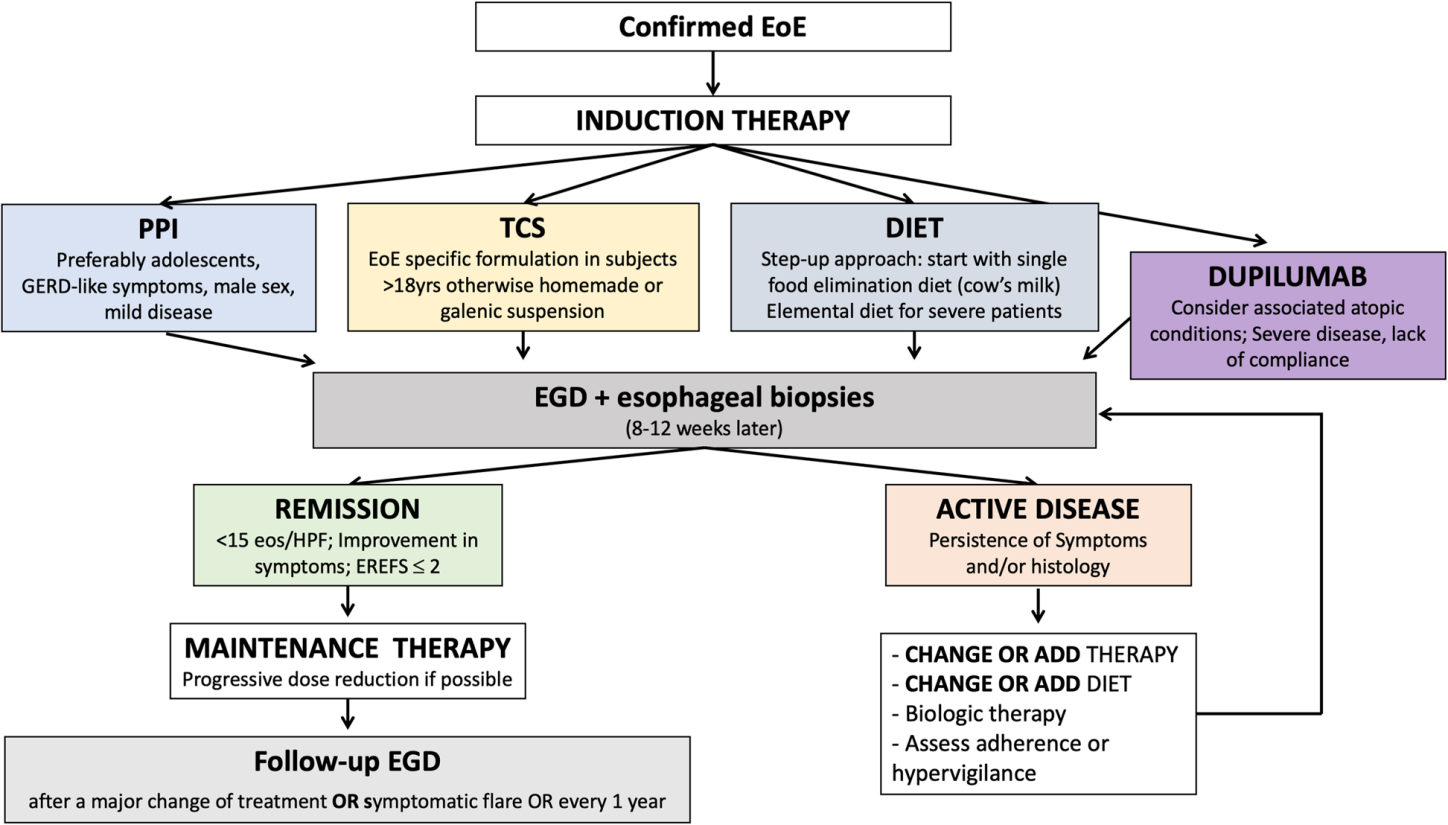
Therapeutic Pyramid for Pediatric EoE. Hierarchical treatment strategy from first-line options (PPIs, diet) to advanced therapies (biologics, dilation), based on disease severity and phenotype

A comprehensive algorithm outlining therapeutic and dietary options for EoE management is presented in Fig. 2, while Table 1 provides a summary of dosages and maintenance strategies for all therapeutic options.

**Table 1:** Treatment and Dosage Guide for Pediatric EoE

Treatment	Induction Dosage	Maintenance Dosage
Proton Pump Inhibitors (PPIs)	2 mg/kg/day divided into 2 doses for 8–12 weeks	1 mg/kg/day once daily
Topical Corticosteroids (Budesonide)	< 10 years or < 150 cm: 1 mg/day; > 10 years or > 150 cm: 2 mg/day	Lowest effective dose after remission
Topical Corticosteroids (Fluticasone)	880–1760 mcg/day divided into 2–4 doses	Lowest effective dose after remission
Single-Food Elimination Diet	Eliminate cow’s milk	Adjust diet based on response
4-Food Elimination Diet	Eliminate milk, wheat, egg, and soy	Adjust diet based on response
6-Food Elimination Diet	Eliminate milk, wheat, egg, soy, nuts, and seafood	Adjust diet based on response
Elemental Diet	Use amino acid–based formula	Maintain if needed; highly restrictive
Biologic Therapy (Dupilumab)	Weight-based dosing: • 15 to < 30 kg: 200 mg every 2 weeks • 30 to < 40 kg: 300 mg every 2 weeks • ≥ 40 kg: 300 mg every week	As the induction dose
Endoscopic Dilation	Performed in case of strictures; not a primary treatment	Not applicable

Beyond pharmacologic and dietary therapy, multidisciplinary management is essential. Given the complexity of EoE and its frequent association with atopic diseases and nutritional challenges, a multidisciplinary approach involving pediatric gastroenterologists, allergists, dietitians, and primary care pediatricians is strongly recommended. To facilitate practical implementation, integrated care models should be promoted, ideally through structured collaboration within dedicated multidisciplinary clinics or coordinated virtual care teams. Strategies to enhance interdisciplinary communication include regular multidisciplinary meetings (e.g., monthly case conferences), shared access to patient electronic medical records, and standardized referral protocols. Practical examples include establishing a “joint EoE clinic” where gastroenterologists and allergists assess patients together, or implementing “rapid referral pathways” where dietitians are automatically involved after diagnosis to support dietary management. In smaller centers, virtual multidisciplinary boards or written shared care plans between specialists and primary care providers can optimize continuity of care. Clear communication with families, coordinated scheduling of consultations, and provision of unified educational resources can further strengthen the effectiveness of multidisciplinary care and improve both clinical outcomes and patient satisfaction.

Equally important is structured patient and family education to ensure long-term treatment success. Families should be provided with clear, accessible information regarding the chronic nature of EoE, treatment options (including medications, dietary strategies, and biologics), and the importance of long-term monitoring even in the absence of symptoms. To improve adherence, it is crucial to set realistic expectations about therapy goals (symptom control, histologic remission, prevention of fibrosis) and the need for repeated endoscopies. Practical tools include providing structured educational handouts, visual aids explaining endoscopic and histologic findings, and encouraging families to maintain a “treatment diary” to track symptoms, medications, and dietary changes. Specific counseling strategies, such as motivational interviewing, can help address barriers to adherence by exploring patient and caregiver concerns, especially around dietary restrictions or medication use. Whenever possible, a multidisciplinary educational session involving gastroenterologists, allergists, and dietitians should be offered shortly after diagnosis. Key messages should be reinforced at each follow-up visit. Furthermore, written individualized care plans and direct access to a care coordinator or nurse can facilitate communication and empower families to actively participate in disease management.

### Quality of life and transition

Q10.1: How is health-related quality of life (HRQoL) defined and best assessed in children with EoE and their parent proxies?

Statement 10.1.1

HRQoL in children with EoE and their parent proxies can be accurately assessed using validated questionnaires.

[low quality of evidence]

[Vote result: Strongly agree: 48%; agree: 52%, neutral: 0%, disagree: 0%, strongly disagree: 0%]

Statement 10.1.2

According to recent available literature, symptoms severity, biological disease activity and dietary therapy may influence quality of life in patients with EoE.

[low quality of evidence]

[Vote result: Strongly agree: 48%; agree: 52%, neutral: 0%, disagree: 0%, strongly disagree: 0%]

Statement 10.1.3

HRQoL measured with validated questionnaires correlates inversely with clinical and histological disease activity and the use of an elimination diet.

[low quality of evidence]

[Vote result: Strongly agree: 48%; agree: 52%, neutral: 0%, disagree: 0%, strongly disagree: 0%]

Recommendation 10.1.1

We suggest using PedsQL to measure QoL in children with EoE as the most reliable and validated method.

[low quality of evidence, grade of recommendation: weak]

[Vote result: Strongly agree: 68%; agree: 12%, neutral: 20%, disagree: 0%, strongly disagree: 0%]

Recommendation 10.1.2

We suggest assessing HRQoL at least at diagnosis, and before transition to adult care.

[low quality of evidence, grade of recommendation: weak]

[Vote result: Strongly agree: 68%; agree: 12%, neutral: 20%, disagree: 0%, strongly disagree: 0%]

Practice Point

HRQoL in young children at the time of EoE diagnosis should be measured alongside parental assessments, despite the risk of parents underestimating symptom burden.

Summary of Evidence:

HRQoL is significantly impacted in children with EoE, affecting their social and psychological well-being [206, 207]. While adult patients often prioritize symptom relief and quality of life as key treatment goals, there is limited research on these aspects in children [208]. Barriers to treatment include steroid side effects and the challenges of elimination diets [209]. A specific EoE module of the PedsQL™ questionnaire has been developed for different age groups and their parents, demonstrating worse HRQoL in children with active disease [210]. Studies suggest that children on elimination diets tend to have lower HRQoL than those receiving swallowed steroids [211]. Therefore, incorporating validated HRQoL questionnaires into EoE treatment assessments is essential [212].

Q10.2: Which model of Health Care Transition should be adopted for adolescents/young adults with EoE?

Statement 10.2

Despite the concept of transition of care is gaining importance and has been reaffirmed for many chronic gastrointestinal diseases (i.e. liver transplantation, Inflammatory Bowel Disease, Cistic fibrosis), there is no standard model for adolescents/young adults with EoE.

[low quality of evidence]

[Vote result: Strongly agree: 48%; agree: 52%, neutral: 0%, disagree: 0%, strongly disagree: 0%]

Practice Point:

- *Health care transition should be introduced in early adolescence, but details and timing of transition should be individualized.*
- *Transition protocol should be established by involving both pediatric and adult gastroenterologists*

Summary of evidence

Dellon et al. describe limiting issues may both involve patients (disease awareness, family resources, familial acceptance of the need for an adult provider), providers (communication between providers, providers with specific interest for health care transition) or the health care system by itself (staff with specific interest for EoE, institutional support, multidisciplinary involvement, presence of a dedicated transition coordinator) [213, 214]. According to the model they propose, the concept of health care transition should be introduced in early adolescence, but details and timing of transition should be individualized [213]. Transition readiness assessment with standard tools is crucial, but specific evaluation tools are warranted for EoE, taking those available for other gastrointestinal chronic diseases [215]. The presence of a coordinator to facilitate the interaction between the patient and the adult provider is also emphasized to ensure full integration in adult clinics, although the figure is often lacking among European realities [216]. This interesting dynamic and step-by-step model should however be tested and validated with specific studies.

### Future research directions

Despite important advances in the understanding and management of EoE, several key research gaps persist that limit the optimization of diagnosis and treatment, especially in pediatric populations. The gold standard for EoE diagnosis remains invasive endoscopy with biopsy, and no non-invasive biomarker has yet demonstrated sufficient sensitivity and specificity to replace histology. Promising minimally invasive tools, such as the esophageal string test and Cytosponge, are under investigation, but require further validation in large, pediatric-specific, longitudinal studies [61, 62]. Similarly, serum biomarkers like eotaxin-3 and periostin show potential for disease monitoring but lack established thresholds and clinical integration pathways. The use of esophageal impedance measurements and EndoFLIP technology to assess mucosal integrity and remodeling is promising but remains investigational, with unclear pediatric applicability [143].

Another major research need is the refinement of predictive markers for disease progression and therapeutic response. Current histologic and endoscopic scoring systems (e.g., EREFS, EoE-HSS) correlate imperfectly with symptom burden and long-term outcomes. Integration of molecular, genomic, and microbiome-based data into personalized models of disease behavior remains an unmet goal. Additionally, the role of the esophageal microbiome in modulating inflammation and barrier function is poorly understood and represents an exciting field for future investigation [35].

In parallel, several ongoing and upcoming clinical trials are exploring both diagnostic innovations and new therapeutic approaches. Among emerging diagnostic strategies, the esophageal string test and Cytosponge are being evaluated in longitudinal studies for their potential to reduce reliance on repeated endoscopy. Mucosal impedance measurements are also under investigation as surrogate markers of disease activity and healing. On the therapeutic front, the approval of dupilumab, an IL-4/IL-13 pathway inhibitor, has paved the way for new biologic strategies targeting key inflammatory pathways in EoE. Trials are currently underway assessing agents such as cendakimab, an IL-13 inhibitor, and benralizumab, an anti-IL-5 receptor monoclonal antibody. Early-phase investigations are also evaluating inhibitors of thymic stromal lymphopoietin (TSLP) and Janus kinase (JAK) pathways. In addition, research into epithelial barrier repair, including agents targeting the CAPN14 pathway, and microbiome-modulating therapies such as synbiotics, may offer novel options for future management. Notable clinical trials as of 2025 are addressing various targets, including IL-13 and IL-5 pathways, non-invasive esophageal sampling devices, epithelial barrier repair mechanisms, and microbiome-based interventions. Importantly, many of these studies include pediatric populations, aiming to extend therapeutic advances to children with EoE.

Although significant progress has been made, bridging the gap between research innovations and everyday clinical practice will require multicenter collaborations, standardized outcome measures, and a strong emphasis on pediatric-specific research.

## Conclusions

This guideline represents the first Italian consensus document dedicated exclusively to the management of pediatric EoE. By combining the expertise of pediatric and adult gastroenterologists, it provides a unified, practical, and evidence-based framework for the diagnosis and treatment of EoE in children and adolescents. Its recommendations reflect the latest scientific advances and emphasize the importance of multidisciplinary care, standardization of diagnostic and follow-up protocols, and individualized therapy selection. As the incidence of EoE continues to rise, these guidelines are essential for improving patient outcomes and promoting equitable access to specialized care throughout Italy.

## Data Availability

Not applicable.

## Abbreviations

AIGO: Italian Association of Hospital Gastroenterologists and Endoscopists
EoE: Eosinophilic Esophagitis
EREFS: EoE Endoscopic Reference Score
EoEHSS: EoE Histology Scoring System
ESPGHAN: European Society for Pediatric Gastroenterology, Hepatology and Nutrition
FLIP: Functional Lumen Imaging Probe
GRADE: Grading of Recommendations, Assessment, Development and Evaluation
HRM: High-Resolution Manometry
IBD: Inflammatory Bowel Disease
MII-pH: Multichannel Intraluminal Impedance-pH Monitoring
PICO: Population, Intervention, Comparator, Outcome
PPI: Proton Pump Inhibitor
SIGENP: Italian Society of Pediatric Gastroenterology Hepatology and Nutrition
SIGE: Italian Society of Gastroenterology
SIED: Italian Society of Digestive Endoscopy
TCS: Topical Corticosteroids
TNE: Transnasal Endoscopy
WG: Working Group
